# Risk factors associated with diarrhea in Danish commercial mink (*Neovison vison*) during the pre-weaning period

**DOI:** 10.1186/s13028-017-0312-1

**Published:** 2017-06-29

**Authors:** Julie Melsted Birch, Jens Frederik Agger, Christina Dahlin, Vibeke Frøkjær Jensen, Anne Sofie Hammer, Tina Struve, Henrik Elvang Jensen

**Affiliations:** 1 Department of Veterinary and Animal Sciences, Faculty of Health and Medical Sciences, University of Copenhagen, Ridebanevej 3, 1870 Frederiksberg C, Denmark; 2 National Veterinary Institute, Technical University of Denmark, Kemitorvet, 2800 Kongens Lyngby, Denmark; 3 Kopenhagen Diagnostics, Kopenhagen Fur, Langagervej 60, 2600 Glostrup, Denmark

**Keywords:** “Sticky kits”, “Wet kits”, Wet kit syndrome, “Greasy kits”, Risk factors, Farm level

## Abstract

**Background:**

Pre-weaning diarrhea in mink, also known as “sticky kits”, is a syndrome and outbreaks occur every year on commercial mink farms in all mink producing countries. Morbidity and mortality can be considerable on a farm with huge economic consequences for the farmer as well as compromised welfare for the mink kits. Although efforts have been taken to identify etiologic agents involved in outbreaks, the syndrome is still regarded as multifactorial and recurring problems on the same farms draw attention to management and environmental risk factors. In the pre-weaning period from May to June 2015, a case control study was carried out on 30 Danish mink farms. Data concerning management, biosecurity, hygiene, feed consumption, antibacterial prescription and production efficiency were analyzed.

**Results:**

The proportion of 1-year old females, farm size (total number of females), energy supply per female in the late gestation period, and dogs accessing the farm area were significantly associated with being a case farm. Case farms were prescribed almost twice the amount of antibacterials per gestational unit (female and litter) as in control farms. Farmers on case farms spent significantly more time nursing and treating the animals and experienced more females with mastitis compared to farmers on control farms. No significant differences in cleaning practices or hygienic measures between case and control farms were found and there were no differences in drinking water quality, bedding material, composition neither of color types nor in management regarding litter equalization.

**Conclusions:**

Results from this study showed an association between the occurrence of pre-weaning diarrhea on mink farms and parity profile, farm size and feeding intensity in the gestational period. The access of dogs to the farm area was a significant risk factor, but needs further clarification.

**Electronic supplementary material:**

The online version of this article (doi:10.1186/s13028-017-0312-1) contains supplementary material, which is available to authorized users.

## Background

**Fig. 1:**
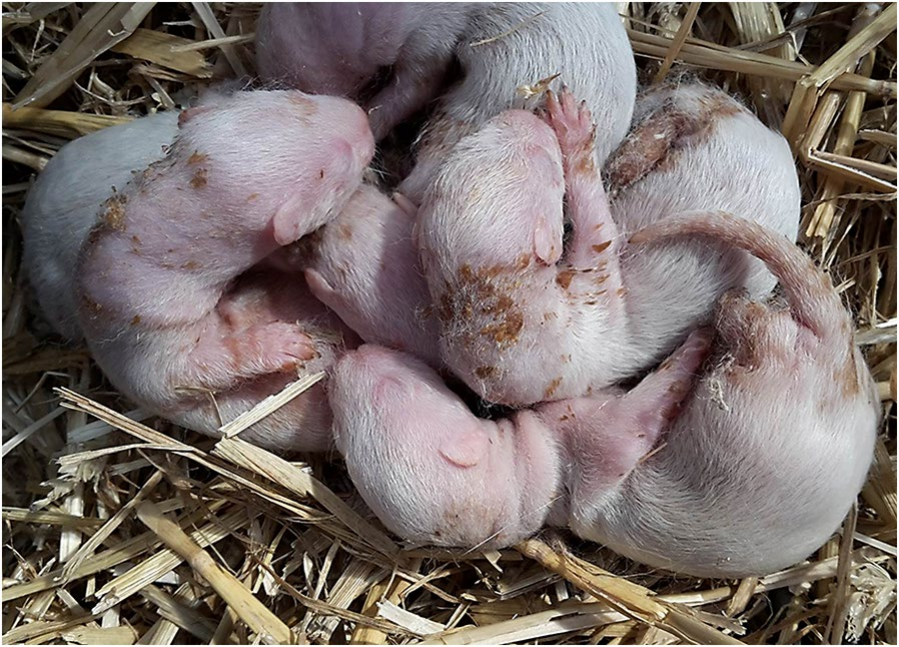
A mink kit litter affected by pre-weaning diarrhea and a greasy/sticky appearance

Diarrhea in the pre-weaning period of mink also known as “sticky kits”, “wet kits” or “greasy kits” is a syndrome, which has been known in Denmark for more than 60 years [1]. Clinical signs are characterized by diarrhea and the appearance of a sticky exudate starting in the neck region, which to a variable extend may spread over the trunk and legs. Greasy, black claws maybe an early sign of the syndrome (Fig. 1). In fulminant, prolonged cases the perineal region becomes edematous and dehydration may develop together with a distressed vocalizing behavior [2].

Whole mink litters are affected and the morbidity rate vary from zero to more than 30% of the litters with a mortality of typically one or two kits per litter [3]. The syndrome is rather common in all mink producing countries although the number of farms and the severity of outbreaks vary from year to year [3]. The economic consequences for the mink farmers can be considerable due to cost for treatment and increased mortality. Efforts have been taken to identify etiologic microorganisms from outbreaks of pre-weaning diarrhea. *Staphylococcus delphini* and *Escherichia coli* have among other bacteria been incriminated as the causes of “sticky kits”. However, mustelids are natural hosts of *S. delphini* group A and it has not been possible to link certain sero-groups of *E. coli* to pre-weaning diarrhea [4–6]. Mink astrovirus (MiAstV) and calicivirus were found to be associated with pre-weaning diarrhea [7] and rota- and coronavirus have been detected in mink kit feces in both healthy and “sticky” kits [5, 8]. Thus, the syndrome is still regarded as multifactorial and different studies have been carried out to identify risk factors in management, feed, milk composition and animals [2]. Henriksen [8] was of the opinion that some feed kitchens were associated with a higher frequency of the syndrome than other feed kitchens, and the morbidity increased with high humidity, temperatures, population density and poor hygiene. In other studies no significant effect of poor hygiene was present, but problems with “sticky kits” were significantly associated with diarrhea later in the growing season, and the problems had a tendency to recur at the farm the following year [9]. Studies on litter level revealed an almost four times increased risk of getting “sticky kits” in young wild type mink females compared to older females [10]. Also large, late born litters and litters from primiparous females were at a greater risk of having “sticky kits” [3, 10]. A higher frequency with pre-weaning diarrhea was seen on large farms compared to small farms and farms with occurrence of pre-weaning diarrhea had a lower feed consumption in late April and recurrent problems in the next season [11, 12]. In addition, females, which had been restricted fed for a prolonged period before birth, had a significant greater risk of having sticky kits/pre-weaning diarrhea in the kits [10]. Individual management practices for the farms include practices of litter equalization, introduction of new breeding dams, hygiene measures as well as biosecurity initiatives, which have not been fully studied. The aim of this study was to identify possible management risk factors on farm level concerning aspects of housing, hygiene, biosecurity and energy supply per female in late gestation for being a farm with outbreak of pre-weaning diarrhea. Secondarily, we also wanted to clarify the prescription patterns of antibacterials and some production efficiency parameters on farms with and without outbreaks of pre-weaning diarrhea.

## Methods

### Study design and participating farms

A case–control study was carried out from the 11th of May to weaning at the end of June 2015 on Danish commercial mink farms (n = 30). The first cases of pre-weaning diarrhea usually occur when mink kits are around two weeks of age, i.e. within the first 2 weeks of May. Since there was no surveillance system for this disease, and because farmers detected cases of diarrhea during their daily work, it was necessary for the investigation team to have pre-arranged agreements with a number of farmers (n = 53). Thus, from this group 15 farms, with a previous history of outbreak of pre-weaning diarrhea, called our investigation team for a visit when onset of pre-weaning diarrhea started, and these farms were designated “expected case farms”. Concurrently, 15 farms, without a previous history of outbreaks of pre-weaning diarrhea, were randomly selected from the group of pre-arranged farms (n = 53) among farms, which received feed from the same feed kitchen in order to eliminate an effect of the feed composition and quality. These farms were designated “expected control farms”. After June 2nd, no more farms contacted us concerning outbreaks. At weaning at the end of June, the percentage of affected litters recorded by the mink farmers was used to determine the final case–control status of the farms. Case farms (n = 14) were defined as farms having 13–77% affected litters during the period, whereas control farms (n = 16) were defined as farms with <8% affected litters. Thus, one expected control farm changed status and became a case farm, whereas two expected case farms appeared to have such limited problems (<8% affected litters), that they were re-categorized as control farms. The rest of the included farms (n = 27) kept their expected status. Since the incidence rate of pre-weaning diarrhea varies from year to year on farms, and the prediction of which farms will be affected is uncertain, sample size calculations were not possible.

### Farm data

Farm data were obtained at visits on the farms and from face-to-face interviews with the mink farmers. The interviews were performed by using standardized questionnaires. Follow-up telephone interviews were made with the mink farmers after weaning. Farm data included: size of farm, production efficiency (fraction of barren females, total number of kits observed after birth, total number of weaned kits), composition of animals (color, age, newly introduced), housing (type of shed, types of litter material, use of wind shield, type of roof on the nest boxes, water source), management (man-hours used on nursing per 1000 females, litter equalization practice, replacement of litter material, use of feed additives, etc.), and hygiene and biosecurity initiatives (cleaning practice between and during seasons, change of clothes and boots for employers and visitors before entrance to the farm, hygiene practice when handling litters, access of dogs and cats to the farm, redistribution of feed left-overs, and flea prophylaxis). The variables farm size, percentage of 1-year old females, and percentage of females with mastitis recorded by the mink farmer were dichotomized. Likewise the color type composition was grouped into light (light and gray) or dark (black and brown). Cleaning of nest boxes and cages was recorded as performed if high pressure washer, water and soap or flaming were used.

### Drinking water analysis

On each farm, a water sample from the drinking water system was collected from the end of two randomly selected mink sheds. The samples were stored, transported and analyzed according to the recommendations by Analyselaboratoriet, Dansk Pelsdyr Foder a.m.b.a., Denmark. The drinking water samples were categorized as above human limit value if one of the samples showed a total germ counts above 200/mL, fluorescence germ counts above 5/mL, coliform bacteria count above 0/100 mL, or thermo stabile coli count above 0/100 mL.

### Feeding

The average feed energy supply per female was based on data from the feed kitchens. It was daily recorded how many kilograms of feed there had been delivered to the farm. The daily delivery in kilograms from week 14 to week 19 (corresponding to the late gestation period) was summed up per week and divided by the total number of animals (females and males if any) on the farm in that particular week. To compensate for differences in energy content in the feed from the supplying feed kitchens, results from regular quality test samples from each kitchen and feed plan were used to calculate the average energy content in a particular week. The results from the test samples were obtained from the Danish Fur Feed [13].

### Antibiotic prescription

In Denmark, each farm has a six digit identity code in the Central Husbandry Register (CHR) [14]. This farm ID was used to merge data from different sources. Data on all prescriptions of antibacterial medicines from March 1 to July 1 2015 were extracted from the national veterinary prescription database, VetStat [15]. Vetstat is considered to cover more than 99% of the total amounts of antibacterials for veterinary use in Denmark (DANMAP 2001) [16]. In VetStat, each prescription is represented by a record, including information on date of purchase, product identity and quantity, farm ID, target animal species, target age group, target disease category, and the identity of the prescribing veterinarian.

All prescribed antibacterials exclusive those for topical use were included. In order to compare amounts of different kinds of antibacterials, the amounts of each antibacterial was converted into number of Defined Animal Daily Doses for treatment of 1 kg of mink (DADD_kg_), as previously described [17].

### Statistical analyses

The data were entered into MS Excel and transferred to SAS Enterprise Guide 7.1 (SAS Institute Inc.2014). Initially the coding and distribution was checked for each variable. Then bivariate analyses of all explanatory variables in relation to the case control status was performed and judged statistically by Student’s t-test (for continuous variables) or Chi square/Fisher’s exact test (for categorical variables) and judged epidemiologically by odds ratio (OR) estimation. Finally, multivariable analyses using the Mantel–Haenszel procedure and logistic analysis for all variables with a P < 0.05 in the bivariate analyses was performed. The reason for only including explanatory variables with a P < 0.05 was due to the small sample size of only 30 farms. The logistic analyses was conducted with proc logistic in SAS with the binary distributed case control status linked to the explanatory variables using the logit function (logit(P) = ln[p/(1−P)]). Model fit was evaluated using the likelihood ratio test. We used manual model-building guided by a causal diagram with evaluation of confounding and interaction. We used the Type 3 Chi square test to evaluate significance of the contribution of each explanatory variable in the model.

## Results

### Descriptive analyses

Farm size varied between 890 and 16,327 females. There were no differences in the percentage of barren females in the two groups, no significant difference in number of kits/female (mated or breeding) and the average number of weaned kits/breeding female in the case group was 5.6 compared to 6.1 in the control group, which was not significant (P = 0.2).

**Fig. 2:**
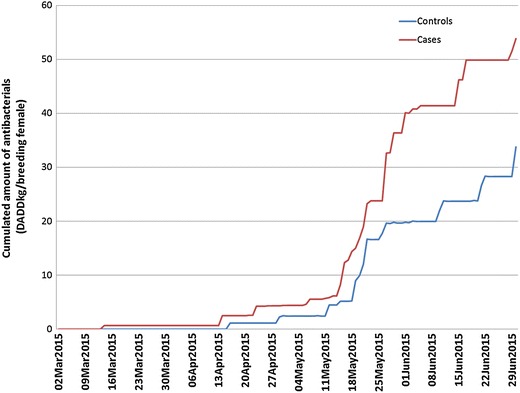
Cumulated amount of prescribed antibacterials for case and control farms

The time used on care and treatment of the mink litters varied significantly. On case farms, 6.1 h/1000 females and on control farms 3.8 h/1000 females were spent (P < 0.01). Figure 2 illustrates the prescription pattern for antibacterials in case farms compared to control farms. Case farms were also prescribed more antibacterials per female (gestational unit) than control farms. The prescription was under 6 DADD_kg_/breeding female for both case and control farms until 11th of May after which an increase was seen for both case and control farms. From 8th of June until weaning the prescription still increases but the rate was somewhat the same between the groups. For the whole period, case farms were prescribed 54 DADD_kg_/breeding female compared to control farms which were prescribed 34 DADD_kg_/breeding female.

### Housing

The risk of being a case farm increased by farm size, i.e. if it was above the median (OR = 5.5, P < 0.05). No differences was found in type of shed, bedding material, type of roof on the nest boxes, use of wind shields on the nest boxes or in the drinking water source (private or public).

### Composition of animals

**Fig. 3:**
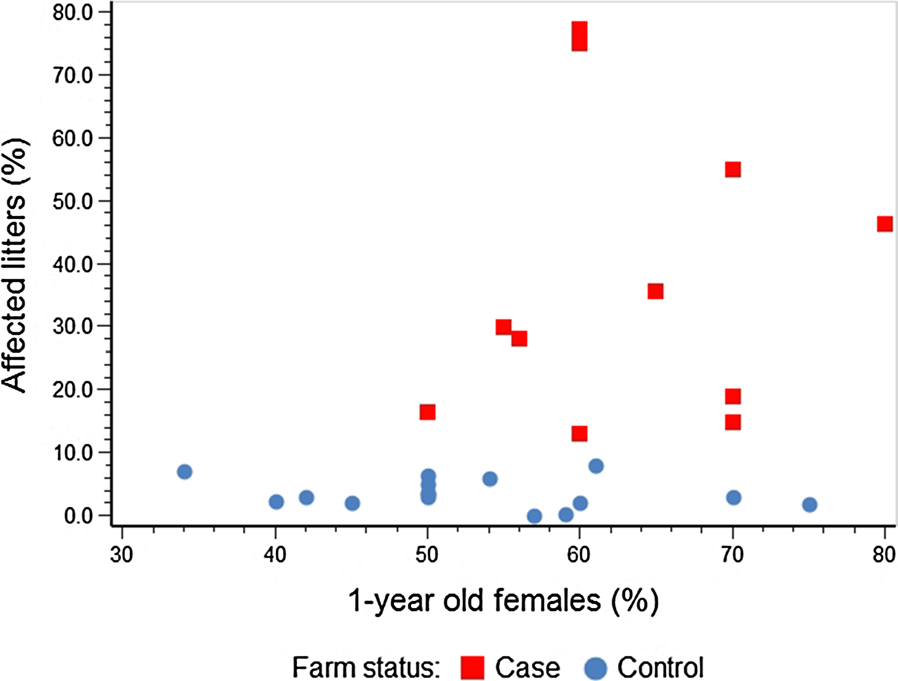
Association between pct. 1-year old females and pct. affected litters with case control status

Figure 3 illustrates the distribution of 1-year old females in relation to the frequency of affected litters. The odds of being a case farm increased with increasing proportion of 1-year old females (OR = 5.9, P < 0.05). There were no differences in the proportion of newly introduced animals or in the color compositions of the animals.

### Feeding

**Fig. 4:**
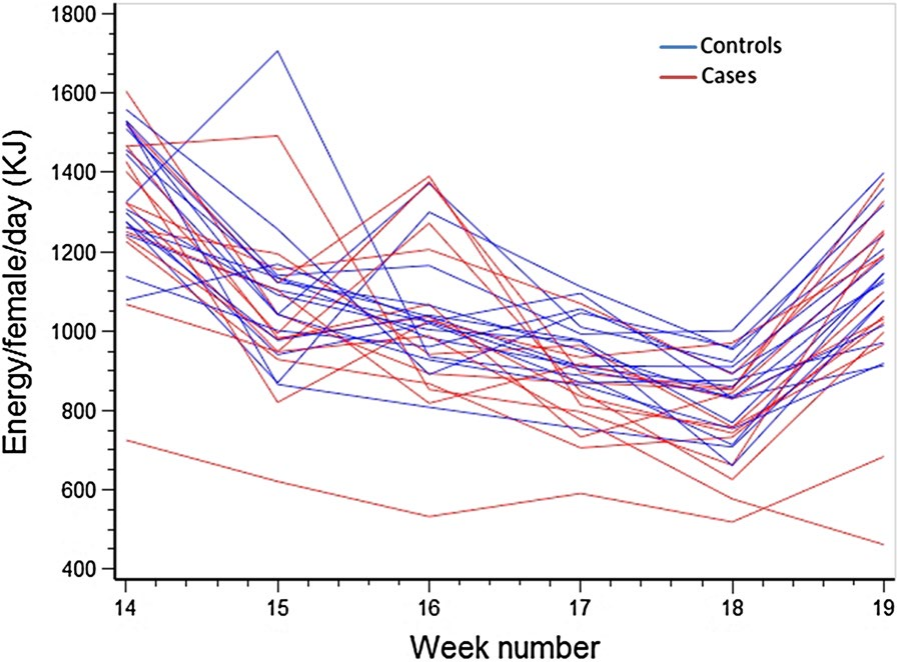
Energy supply per female in April and May 2015 for case and control farms

The average weekly energy delivered per female was lower in week number 17 in the case farms (839 kJ) compared to the control farms (957 kJ) (P < 0.01). In week number 18, the females on case farms received 758 kJ compared to females on control farms which received 842 kJ and the difference was significant (P = 0.05). In all the other weeks, females on case farms received less energy than females on control farms, but the difference was not significant. There was a tendency of general declining energy supply in the late gestation from week 14 to week 18 and then it increased again in week 19. Figure 4 shows the energy supply per female on a weekly basis and mean values are listed in Table 1.

**Table 1:** Feed energy supply per mink female per day (kJ)

Period	Case farms (n = 14)	Control farms (n = 16)
Week 14	1307 ± 219	1360 ± 150
Week 15	1023 ± 198	1099 ± 194
Week 16	1019 ± 235	1034 ± 143
Week 17**	839 ± 119	957 ± 95
Week 18*	758 ± 128	842 ± 99
Week 19	1063 ± 248	1139 ± 145

Mean values ± standard deviation

** P < 0.01; * P = 0.05

The accumulated energy supply per female from week 14 to week 19 was 42,074 ± 5970 and 45,025 ± 3156 kJ for case and control farms, respectively (P = 0.11). The accumulated energy supply per female in week 17–18 was significantly lower (P < 0.01) in case farms compared to control farms with an accumulated energy per female of 11,183 ± 1580 and 12,597 ± 1185 kJ, respectively, for case and control farms.

### Management

No difference in how litter-equalization was used between case and control farms was found. Moreover, no difference was seen in how the mink farmers changed the nest bedding in healthy and diseased litters. None of the farms used water supplementation on the nest boxes during the early pre-weaning period or at the time of pre-weaning diarrhea.

### Hygiene

Dogs’ access to the farm area increased the risk (OR = 9.3, P < 0.05) of being a case farm. No associations were revealed in hygiene precautions such as use of boot cover, changing clothes before entering the farm or use of gloves or hand disinfectant between handling of mink litters in case farms compared to control farms. Neither could an association between farm status and the practice of redistribution of feed left-overs be seen. Drinking water quality was not a risk factor for farm status, and no association between flea prophylaxis and farm status was found.

The bivariate analyses are shown in detail in Additional file 1.

### Multivariable analyses

**Fig. 5:**
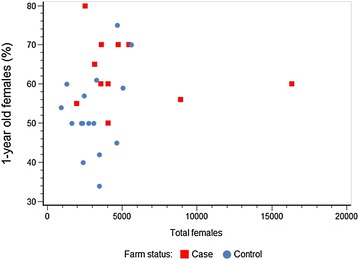
Association between farm size and pct. 1-year old females with case control status

Farm size was associated with the proportion of 1-year old females (OR = 5.6, P < 0.05) and large farms had an increased proportion of 1-year old females compared to small farms (Fig. 5). Hence, farm size was a confounder for the proportion of 1-year old females. Adjusted for farm size by Mantel–Haenszel analysis still gave a positive OR (=3.7), but it was no longer significant (P = 0.32).

**Fig. 6:**
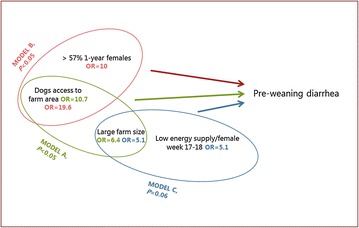
Multivariable models for risk factors associated with case control status of pre-weaning diarrhea

The following four risk factors (1) access of dogs to the farm area (yes/no), (2) farm size (>/< median of 3461 females), (3) proportion of 1-year old females (>/<57%) and (4) the accumulated energy supply per female in weeks 17–18 (>/<12,196 kJ) were chosen to multivariable analysis by logistic regression. Two models showed to be significant (P < 0.05) and one nearly significant (P = 0.06) (Fig. 6). In Model A comprising farm size and dogs’ access to the farm area, the odds of being a case farm were higher for large farms compared to small farms, given dogs’ access to the farms were held constant (OR = 6.4, P < 0.05). The odds of being a case farm were also higher for farms with dogs’ access to farm area compared to farms, where dogs were not allowed to enter, (OR = 10.7, P < 0.05) if farm size were held constant. In model B comprising proportion of 1-year old females and dogs’ access to the farm area, the odds of being a case farm increased if the farm had more than 57% 1-year old females compared to farms having less than 57% 1 year old females (OR = 10, P < 0.05), given dogs’ access were held constant. The odds of being a case farm increased also if dogs were allowed access compared to farms where dogs didn’t have access to the farm area (OR = 19.6, P < 0.05), given the proportion of 1-year old females were held constant. Model C comprised farm size and accumulated energy supply per female in weeks 17–18. There was an almost significant effect of farm size (OR = 5.1, P = 0.06) given the accumulated energy supply in weeks 17–18 were held constant. Farms supplying less than 12,196 kJ per female in weeks 17–18 also had a nearly significant increase in odds of being a case farm compared to farms giving more than 12,196 kJ per female (OR = 5.1, P = 0.06), when farm size were held constant.

## Discussion

The case–control status could be explained by three multivariable models. A single model with more than two variables was not possible to fit due to too few observations.

Dogs’ access to the farm area contributed to two of the models, which may reflect that dogs are carriers of microorganisms involved in the pre-weaning diarrhea syndrome. Another explanation is that dogs’ access to the farm area was a proxy for other management of biosecurity and hygiene, meaning that farmers allowing dogs on the farm had a more relaxed approach to biosecurity and hygiene in general. Farm size was a risk factor in two of the multivariable models, which was also found in a study of pre-weaning diarrhea in pigs [18]. However herd size and herd density can be measured in different ways and the primary factor that produces the herd size effect may be found in management and environmental factors and can be protective as well [19]. The proportion of 1-year old females on the farm was a significant risk factor in bivariate analysis as well as in the multivariable analysis. This finding is in line with previous studies conducted on litter level [3, 10]. This might be explained by poorer nursing skills in the primiparous females or a poorer passive immunity transfer from young females to the kits compared to older females. A Norwegian study in dairy cows showed that cows in their fourth parity or more had significantly higher levels of IgG in the colostrum compared to cows in their first or second parity, and this might also be the case in mink [20]. Interestingly, the proportion of 1-year old females was significantly associated with farm size. Large expanding farms with ambitions of fast phenotypic improvement will have a consequential high female turnover which may be an explanation for this association. Thus, a high proportion of 1-year old females and a high morbidity rate will as a consequence prevent the mink farmers from accomplish a breeding strategy with selection on disease free animals including females with the best colostrum quality. The accumulated energy delivered per female in weeks 17 and 18 was significant in relation to case control status and corresponds to a daily supply of 799 kJ per female per day and 900 kJ per female per day for case and control farms, respectively. Hyperleptinemia in the pregnant mink may exert anorexigenic effects in the second but especially the last trimester resulting in a declining energy uptake and negative energy balance [21]. Some of the general declining energy consumption in the last gestation period is therefore due to a natural decline in appetite among the females. However there is no obvious reason why females on case farms should consume less than females on the control farms. Therefore, the mink farmers had restricted the minks’ energy intake, perhaps in an attempt to get more active females prior to birth. The results is supported by other studies in which restricted feeding during the gestation period was found to be a risk factor for pre-weaning diarrhea in mink kits [10, 22]. Based on these studies, a critical limit of 879 kJ per female per day was suggested [23]. With considerable variation in weather, quality of insulation in nest boxes and management on the farms added to a tendency of increasing animal size, energy consumption and safety limits should continuously be evaluated. Mastitis as a triggering factor for “sticky kits” has been suggested [8].

Mastitis was indeed a concurrent problems in many of the farms, however mastitis was not found to be a necessary cause for the development of “greasy kits” [24]. Incidences of mastitis above 10% was a risk factor of being a case farm, but diagnosing “mastitis” was mainly done by the staff on the farm when appetite in the female ceased and imply difficulties owing to the fact that mastitis in mink often is subclinical [24]. Mastitis in the female may, however, still be a risk factor in a multifactorial disease complex, but further studies are needed to clarify this. Besides dogs’ access to the farm area, no association to hygiene or biosecurity precautions was found. However, an explanation for the lack of proof for good practice of hygiene and biosecurity could be that the etiologic agents involved in pre-weaning diarrhea is very common in the animals during this period, and the cause should be searched for in a more generalized debility of the females and kits.

The pattern in antibacterial prescription showed that case and control farms were prescribed antibacterials before the onset on pre-weaning diarrhea, and the rapid increase from 11 to 18th of May until 8th of June corresponds very well with the period of outbreaks of pre-weaning diarrhea. The largest difference in prescription rate between the two groups was seen from 25th of May to 8th of June after which time outbreaks of pre-weaning diarrhea usually cease in Denmark [11, 25]. The fact that case farms were prescribed more antibacterials prior to the onset of pre-weaning diarrhea must reflect farm history based expectations of more problems on case farms compared to control farms. Prescription of antibacterials has increased during recent years in Danish mink farms and associated with herd size, season and the laboratory diagnosis of MiAstV [17]. The results from this study suggest that further investigation should focus on young females, and consultants of farms with recurrent problems of pre-weaning diarrhea should pay attention to parity profile and feeding strategies especially on large farms.

## Conclusions

Associations between farm size, parity profile, energy supply in late gestation and farm status was revealed. Also dogs accessing the farm area was a risk factor. The study did not find any other associations in hygiene practices, housing or in management between case and control farms. Antibacterial prescription was higher and staff spent more time nursing and treating the animals on case farms compared to control farms.

## Additional file

**Additional file 1.** Results of bivariate analysis of associations between farm status for pre-weaning diarrhea in mink and explanatory variables.
