# Supplementary material for: Risk factors associated with diarrhea in Danish commercial mink (Neovison vison) during the pre-weaning period
Source: Acta Vet Scand. 2017 Jun 29;59:43. doi: 10.1186/s13028-017-0312-1 (PMC5492706; doi:10.1186/s13028-017-0312-1)
Supplement: Supplementary file 1 — Additional file 1. Results of bivariate analysis of associations between farm status for pre-weaning diarrhea in mink and explanatory variables. [file 13028_2017_312_MOESM1_ESM.docx]

| **Additional file 1. Results of bivariate analysis of associations between farm status for pre-weaning diarrhea in mink and explanatory variables** | | | | | |
| --- | --- | --- | --- | --- | --- |
| **Variable** | **N^a^** | **Cases** | **Controls** | **OR** | ***P* value** |
| **Production efficiency** |  |  |  |  |  |
| Empty females (%) | 30 | 7.9 | 8.0 |  | 0.91 |
| Total number of kits observed after birth/ litter born | 24 | 6.6 | 6.7 |  | 0.88 |
| Number of kits at weaning/parturient female | 27 | 5.6 | 6.1 |  | 0.20 |
| **Health** |  |  |  |  |  |
| Pct. Females with mastitis in lactation period observed by farmer  >10%  <10% | 30 | 4  10 | 0  16 | - | 0.037 |
| **Housing** |  |  |  |  |  |
| Farm size  >median^b^  <median | 30 | 10  4 | 5  11 | 5.5  1 | 0.028 |
| Type of shed  Open  Open and closed  Closed | 30 | 4  8  2 | 10  4  2 | 0.4  2.0  1 | 0.19 |
| Use of wind shield on the nest box from birth  No  Yes | 30 | 3  11 | 0  16 | - | 0.09 |
| Roof of nest boxes  Masonite plate  Straw  Mix | 30 | 2  11  1 | 4  12  0 | 1  1.83  - | 0.65 |
| Drinking water supply  Public  Private | 29 | 3  10 | 8  8 | 0.3  1 | 0.25 |
| Additives to the drinking water  No  Yes | 30 | 11  3 | 13  3 | 0.85  1 | 1.00 |
| Uncut straw in the nest boxes  No  Yes | 30 | 9  5 | 14  2 | 0.26  1 | 0.20 |
| Cut straw in the nest boxes  No  Yes | 30 | 1  13 | 1  15 | 1.15  1 | 1.00 |
| Easy-stroe in the nest boxes  No  Yes | 30 | 1  13 | 3  13 | 0.33  1 | 0.6 |
| Wool in the nest boxes  No  Yes | 30 | 14  0 | 13  3 | - | 0.23 |
| Wood chips in the nest box  No  Yes | 30 | 14  0 | 13  3 | - | 0.23 |
|  |  |  |  |  |  |
| **Variable** | **N^a^** | **Cases** | **Controls** | **OR** | ***P* value** |
| Straw  Wheat  Barley | 30 | 14  0 | 15  1 | - | 1.00 |
| **Animal composition** |  |  |  |  |  |
| New females introduced this year (%) | 30 | 1.9 | 2.3 |  | 0.76 |
| New females introduced in the last 3 years (%) | 29 | 10.4 | 12.2 |  | 0.73 |
| Dark color types (%) | 25 | 51 | 64 |  | 0.24 |
| Light color types (%) | 26 | 46 | 33 |  | 0.23 |
| One-year old females  >57%  <57% | 27 | 8  3 | 5  11 | 5.9 | 0.034 |
| **Management** |  |  |  |  |  |
| Use of feed additive  No  Yes | 29 | 9  4 | 12  4 | 0.75  1 | 1.00 |
| Systematic litter equalization for max. kits/litter  Yes  No | 30 | 12  2 | 13  3 | 1.4  1 | 1.00 |
| Systematic litter equalization for min. kits/litter  Yes  No | 30 | 8  6 | 10  6 | 0.8  1 | 0.76 |
| Limit for maximum litter size  8-9  10-12 | 25 | 6  6 | 9  4 | 0.44  1 | 0.56 |
| Limit for minimum litter size  2-3  4-5 | 18 | 6  2 | 8  2 | 0,75  1 | 0.75 |
| Number of females per staff member | 30 | 1112 | 1213 |  | 0.33 |
| Hours spent on nursing and treatment/1000 females^c^ | 29 | 6.1 | 3.8 |  | 0.002 |
| **Hygiene and biosecurity** |  |  |  |  |  |
| Cleaning of cages between seasons  No  Yes | 27 | 3  8 | 2  14 | 2.63 | 0.37 |
| Cleaning of nest boxes between seasons  No  Yes | 28 | 6  6 | 4  12 | 3.00  1 | 0.24 |
| Flea prophylaxis  No  Yes | 30 | 1  13 | 0  16 | - | 0.46 |
| Use of drying agent in the nest boxes  No  Yes | 30 | 12  2 | 13  3 | 1.38  1 | 1.00 |
| Hand disinfectant or use of gloves between healthy and diseased litters  No  Yes | 30 | 8  6 | 12  4 | 0.44  1 | 0.44 |
|  |  |  |  |  |  |
|  |  |  |  |  |  |
| **Variable** | **N^a^** | **Cases** | **Controls** | **OR** | ***P* value** |
| Distribute feed left-overs to other cages  Yes  No | 30 | 7  7 | 9  7 | 0.77  1 | 0.73 |
| Staff change footwear before entrance to farm  No  Yes | 30 | 7  7 | 8  8 | 1  1 | 1.00 |
| Staff change clothes before entrance to farm  No  Yes | 30 | 6  8 | 7  9 | 0.96  1 | 0.96 |
| Staff wash hands before entrance to farm  No  Yes | 30 | 11  3 | 14  2 | 0.52  1 | 0.64 |
| Visitors change footwear before entrance to farm  No  Yes (incl. visitors who only change footwear if they have had contact with fur animals) | 30 | 1  13 | 1  15 | 1.15  1 | 1.00 |
| Visitors change clothes before entrance to farm  No  Yes (incl. visitors who only change clothes if they have had contact with fur animals) | 30 | 7  7 | 10  6 | 0.6  1 | 0.71 |
| Visitors wash hands before entrance to farm  No  Yes (incl. visitors who only wash hands if they have had contact with fur animals) | 30 | 12  2 | 15  1 | 0.4  1 | 0.58 |
| Cats have access to farm area  Yes  No | 30 | 4  10 | 10  6 | 0.24  1 | 0.06 |
| Dogs have access to farm area  Yes  No | 30 | 8  6 | 2  14 | 9.33  1 | 0.019 |
| **Drinking water quality^d^** |  |  |  |  |  |
| Total germ count  Above limit  Within limit | 30 | 12  2 | 12  4 | 2.00  1 | 0.66 |
| Fluorescent germ count  Above limit  Within limit | 30 | 8  6 | 6  10 | 2.22  1 | 0.28 |
| Coliform bacteria  Yes  No | 30 | 3  11 | 1  15 | 4.1  1 | 0.28 |
| Thermo stabile coli  Yes  No | 30 | 1  13 | 0  16 | - | 0.47 |
|  |  |  |  |  |  |
| **Variable** | **N^a^** | **Cases** | **Controls** |  | ***P* value** |
| **Feed energy (KJ) supply per female per day^e^** |  |  |  |  |  |
| Week 14 | 30 | 1307 ± 219 | 1360 ± 150 |  | 0.45 |
| Week 15 | 30 | 1023 ± 198 | 1099 ± 194 |  | 0.3 |
| Week 16 | 30 | 1019 ± 235 | 1034 ± 143 |  | 0.83 |
| Week 17 | 30 | 839 ± 119 | 957 ± 95 |  | 0.005 |
| Week 18 | 30 | 758 ± 128 | 842 ± 99 |  | 0.05 |
| Week 19 | 30 | 1063 ± 248 | 1139 ± 145 |  | 0.31 |
| Week 17-18 cumulated | 30 | 11183 ± 1580 | 12597 ± 1185 |  | 0.009 |
| Week 14-19 cumulated | 30 | 42074 ± 5970 | 45025 ± 3156 |  | 0.11 |
| ^a^ Numbers of farmers that answered the question  ^b^ median=3461 females  ^c^ may be an effect  ^d^ Limit values: Total germ count (max 200/ml), Fluorescent germ count (max. 5/ml), Coliform bacteria (max 0/100ml), Thermo stabile coli (max. 0/100 ml)  ^e^Mean value ± standard deviation | | | | | |
